# A Case of Rapidly Growing Tricuspid Valve Papillary Fibroelastoma Presenting With Syncope

**DOI:** 10.7759/cureus.55510

**Published:** 2024-03-04

**Authors:** Ibrahim Kamel, Harold Dietzius, Toni Magee, Sadaf Esteghamati

**Affiliations:** 1 Internal Medicine, Tufts Medical Center, Boston, USA; 2 Internal Medicine, Steward Carney Hospital, Dorchester, USA; 3 Cardiology, Steward Carney Hospital, Dorchester, USA; 4 Internal Medicine, University of La Verne, La Verne, USA

**Keywords:** cardio-oncology, cardiac imaging, syncope, papillary fibroelastoma, tricuspid valve

## Abstract

Cardio-oncology, at the intersection of cardiovascular diseases, oncological conditions, and treatments, presents unique challenges in medical care. This abstract highlights a case involving a 60-year-old male presenting with syncope at work; the workup revealed a rapidly growing tricuspid valve papillary fibroelastoma (PFE), emphasizing diagnostic approaches, management strategies, and clinical implications. The diagnostic investigation, including blood cultures, transthoracic echocardiogram, transesophageal echocardiogram, and cardiac MRI, confirmed the diagnosis of tricuspid valve PFE. A multidisciplinary approach led to a shared decision with the patient to opt for serial monitoring. Syncope was attributed to dehydration. This case underscores the complexities of managing cardiovascular conditions in the context of oncology and the importance of collaborative decision-making in patient care.

## Introduction

Papillary fibroelastomas (PFE) represent a rare but noteworthy subset of benign cardiac tumors, predominantly afflicting the valvular endocardium, particularly favoring the left-sided valves. Despite their infrequency, they hold significance in clinical practice, constituting approximately 11.5% of all primary cardiac tumors and often manifesting with embolic complications, particularly prevalent among middle-aged individuals [1]. These tumors exhibit a characteristic slow growth, with an average annual rate of 0.5 ± 0.9 mm/year, and display a diverse range in size, spanning from 2 mm to 70 mm, with a mean diameter of 9 mm [2]. They commonly appear pedunculated on echocardiography, showcasing autonomous movement and a distinct speckled pattern along their edges. Their origin may stem from either the aortic or ventricular surface of the valves. In the realm of treatment, options primarily entail surgical excision or serial observation, with surgery typically recommended unless contraindicated. The overarching goal of surgical intervention is the complete removal of visible tumor tissue while addressing any resultant intracardiac abnormalities through reconstruction. Notably, excision of PFEs can be conducted safely, aiming to preserve the integrity of the original valve and mitigate neurological complications. Both immediate surgical outcomes and long-term prognosis following fibroelastoma removal showcase remarkable success, underscoring the importance of understanding and effectively managing this intriguing cardiac entity [3].

## Case presentation

A 60-year-old male experienced syncope at work, resulting in a head injury. He quickly regained consciousness and was transported to the emergency department.

Medical history

Hypertension was noted. The last echocardiography was unremarkable (five months prior).

Workup

The patient was assessed to determine the cause of syncope. A head CT scan was unremarkable for any acute intracranial abnormalities. A chest computed tomography angiography ruled out pulmonary embolism. Carotid Doppler was also conducted, which returned negative for stenosis.

**Figure 1:**
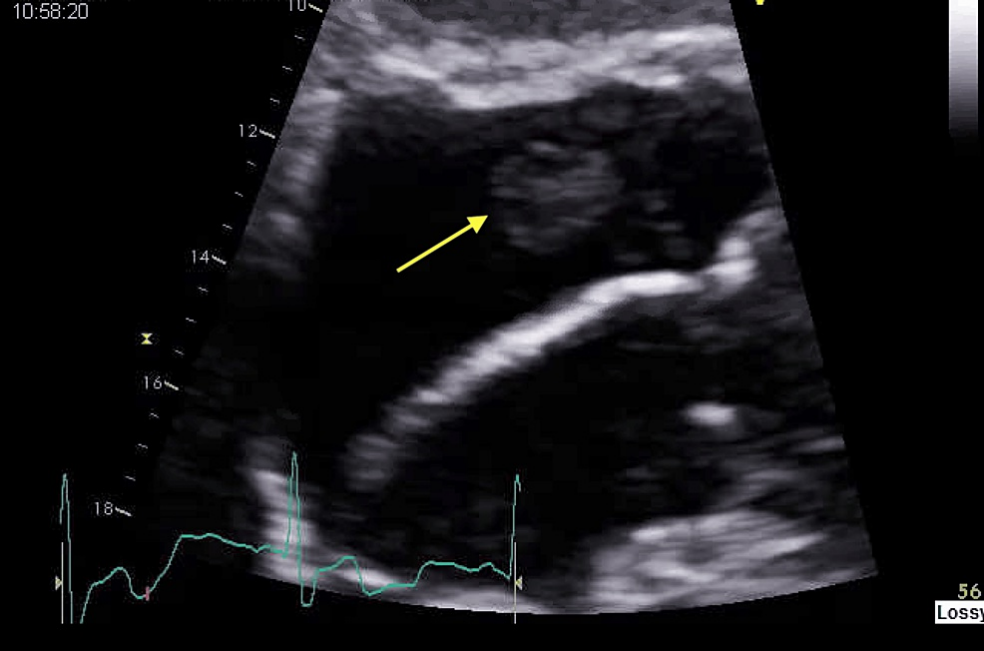
2D echo A large (1.6 x 1.7 cm) round echo dense mass detected on the atrial side of the tricuspid valve is indicated by a yellow arrow in the TTE image. TTE: Transthoracic echocardiogram; 2D: Two-dimensional

Further investigation through a transthoracic echocardiogram (TTE) revealed a mass on the tricuspid valve (Figure 1).

**Figure 2:**
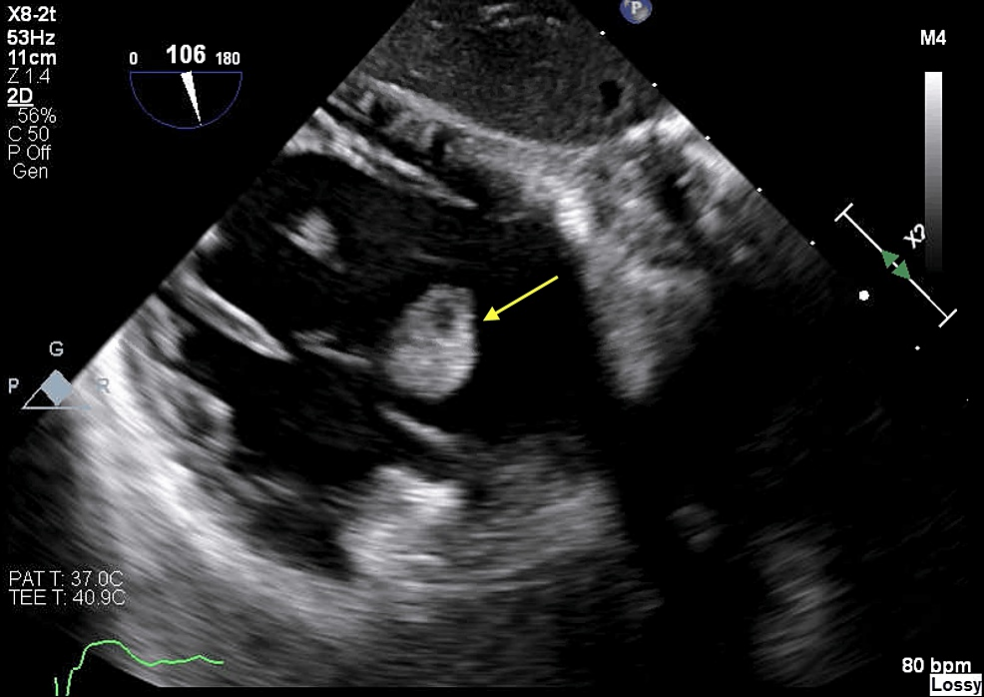
Transesophageal echocardiogram A large (1.6 x 1.7 cm) round echo dense mass observed on the atrial side of the tricuspid valve is highlighted by a yellow arrow in the TEE image. TEE: Transthoracic echocardiogram

However, concerns about infective endocarditis were dismissed as blood cultures returned negative, and no risk factors such as IV drug use were identified. Subsequent evaluation through a transesophageal echocardiogram (TEE) provided more detailed insights, revealing a 16 x 17 mm mobile echo density on the anterior leaflet of the tricuspid valve (Figure 2).

**Figure 3:**
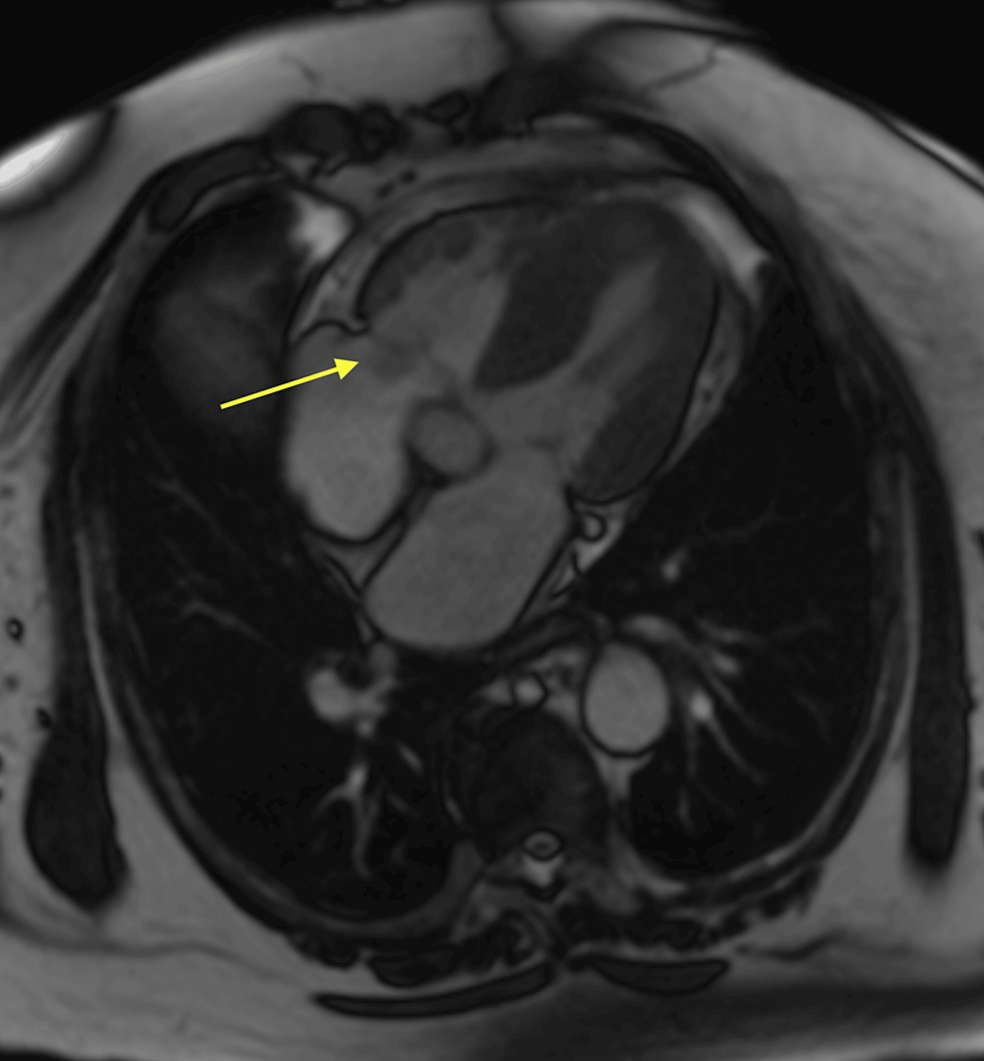
CMR A mass attached to the tricuspid valve is indicated by a yellow arrow. CMR: Cardiovascular magnetic resonance

The cardiovascular magnetic resonance (CMR) examination confirmed a 15 x 11 mm mass attached to the atrial aspect of the tricuspid valve, displaying characteristic features indicative of PFE. These features include peri-lesional flow artifact, homogeneous late gadolinium enhancement (LGE), and T1 and T2 weighted images showing a fibroelastic composition with uniform intermediate signal intensity similar to the myocardium (Figure 3).

Management

Surgical excision is the recommended treatment for larger left-sided PFEs due to the associated risk of embolic complications. However, conservative management of right-sided PFEs can be advised unless they cause hemodynamically significant obstruction or pose a risk of paradoxical embolism. Due to the large size and growth rate, surgical intervention was advised.

## Discussion

In our case, the patient exhibited a rapidly growing mass on the tricuspid valve. PFE had not been observed in the previous TTE performed five months before. The initial study was technically difficult due to obesity and body habitus. Still, given the reported sensitivity and specificity of TTE for the detection of PFE ≥2 mm of 88.9% and 87.8%, respectively, it is unlikely that the PFE had been overlooked at that time [4], suggesting, in turn, the high possibility that it had grown to 17 mm over the course of five months. The tumor's distinctive features include a small size, independent motion, and attachment to an endocardial surface. The TEE may reveal stippled or shimmering borders due to a tumor-blood interface vibration. CMR further aids in characterizing the tumors by demonstrating a lack of contrast uptake in the first-pass perfusion. However, there is a circumferential enhancement in delayed imaging, as observed in this case.

## Conclusions

The case emphasizes the critical role of thorough cardiovascular assessments in patients with syncope, particularly in detecting rare conditions like PFEs. With advancements in cardiac imaging, these tumors are now more detectable, warranting a heightened awareness among healthcare providers. Early identification and intervention not only enhance patients' quality of life but also mitigate the morbidity and mortality risks associated with such tumors. Timely recognition of rapid valvular mass growth underscores the necessity for prompt intervention to prevent severe complications. Shared decision-making between healthcare providers and patients becomes paramount, considering the potential consequences of conservative management versus intervention. While pathology confirmation was deferred by the patient, the imperative for vigilant monitoring and proactive management remains crucial in optimizing patient outcomes and averting adverse events.
